# The Contagion of Cancer

**Published:** 1907-08-03

**Authors:** 


### The Hospital

A Journal of

The Medical Sciences and Hospital Administration.

New Series. No. 19, Vol. I. [No. 1091, Vol. XLII.] SATURDAY, AUGUST 3, 1907.

### THE CONTAGION OF CANCER.

In his address in surgery delivered at the meeting of the British Medical Association at Exeter Mr. Henry Butlin once again comes forward as a supporter of the view that cancer is a communicable disease. Mr. Butlin has already championed this cause in the Bradshaw lecture of 1905, but he now approaches the question from a somewhat different standpoint. At the outset he lays down certain postulates that must be satisfied before any case of cancer can be regarded as showing reliable evidence of contagion. Firstly, the two growths must be of the same variety; thus a squamous cancer can only give rise by contagion to another squamous cancer, and not to a rodent ulcer or to a columnar-celled growth. In the next place, the affected parts must be from time to time in actual contact. Finally, all diagnoses must be confirmed by a microscopical examination. Moreover, he points out that contagion can scarcely arise from a "covered" cancer, that is to say, a cancer buried deeply in the tissues, or clothed by healthy skin; therefore, all such cases must be accepted with great reserve.

It is evident that a close adherence to these criteria will greatly restrict the possibility of contagion, and Mr. Butlin thinks that this must be of rare occurrence, except in the case of what he calls "auto-inoculation." By this term he denotes the appearance of two or more cancers in contiguous parts of the body of the same individual. If the occurrence of auto-inoculation can be satisfactorily proved, "there is," he maintains, "a *prima facie* case for contagion." He gives an outline of fifteen cases by way of illustration. In five of these the cancers appeared on the opposed surfaces of the vulva, in four on the vocal chords, in one on the upper and lower lip. Some of these cases are very striking. They fall in with the conditions set forth at the commencement, and seem to yield more readily to Mr. Butlin's explanation than to any other. He believes that by means of them the case for auto-inoculation is proved, and states his intention of passing on to the study of the communicability of cancer from one individual to another. This, as he says, will be a work of great difficulty, in that the strict conditions so rightly imposed exclude almost all the few known instances of cancer *à deux*.

Let us now examine Mr. Butlin's proposition in greater detail. He states that the cancer cell is a parasite, and that cancer is contagious within certain limits, at present very narrow. To these statements we are inclined to agree, although by so doing we probably do not imply nearly so much as does their author. We believe the cancer cell to be a parasite in the sense that it lives and multiplies at the expense of its host, its power of growth being bound up in the cell itself, or at any rate not depending on the action of any subsidiary parasite such as a micro-organism. On the other hand, we do not believe the cancer cell to be a parasite in the sense that it is, save in very exceptional instances, introduced into the body from without. In the same way we see no reason to dispute the claim that cancer may on rare occasions be auto-inoculable, a fact to which other observers already have called attention.

The phenomena of auto-inoculation, as described by Mr. Butlin, seem to differ merely in degree from the artificial production of cancer in mice. In the latter case the transplanted cells are securely lodged beneath the skin, whereas in the former they are, presumably, deposited on an exposed surface, whence they may be easily dislodged by any movement of the part, and this may help to explain the rarity of the process. The transplantation of cancer cells from animal to animal is usually regarded as an artificial metastasis, and, although the parallel is not exact, the conditions are very similar save for the slight variation in surroundings which must exist even among individuals of the same species. We may, then, consider the process of auto-inoculation as a type of metastasis, where the chances are all against the invading cells establishing their footing.

Mr. Butlin's observations are interesting, and of importance from a practical point of view, for they show the necessity for early treatment, and also the risk of wound-infection during an operation. But we cannot see that they materially alter the present position of the cancer problem; for they shed no fresh light on either the cause or origin of cancer, except in those very rare cases where the required conditions are fulfilled admitting of the explanation that the disease may be due to contagion. The facts, both new and old, seem best to be met by Ehrlich's recent suggestion that the receptors of cancer cells are endowed with an abnormal avidity for nutrient materials, whereas the general receptor mechanism of the cancer patient is less active than usual. If this be so, cancer must depend on a constitutional as well as on a local defect. Ehrlich also believes that the failure of growth on the part of a transplanted tumour is due to an "atreptic" immunity on the part of the new host; that is to say, to an absence of the required nutritive substances rather than to any active effort of defence. And this, together with the infrequent opportunity, may explain the great rarity of contagion between man and man. We feel it to be a pleasure to draw attention to the moderation and fairness with which Mr. Butlin states his case, contrasting so favourably with the reckless eagerness too often characteristic of those who write on this subject.

